# Milker’s nodule: An under‐reported and under‐diagnosed occupational infection

**DOI:** 10.1002/ccr3.2850

**Published:** 2020-04-14

**Authors:** Guru Prasad Poudel, Sudha Agrawal, Sushil Dhakal

**Affiliations:** ^1^ Department of Dermatology and Venereology B.P. Koirala Institute of Health Sciences Dharan Nepal; ^2^ Department of Pathology B.P. Koirala Institute of Health Sciences Dharan Nepal

**Keywords:** Milker's nodule, occupational dermatosis, paravaccinia, pseudocowpox

## Abstract

Single or multiple minimal painful nodulo‐ulcerative lesions over hand in dairy farm worker suggest likelihood of Milker's nodule.Use of personal protective equipment and antiseptics suffice treatment without unnecessary investigation and medicine.

## Graphical abstract

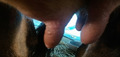

## 1 INTRODUCTION

Occupational dermatosis is described as any change in the mucocutaneous or annexes that is caused, conditioned, maintained, or aggravated by agents present in the occupational activity or working environment.^1^ Milker's nodules is a highly contagious, Zoonotic, self‐limited disease caused by the Paravaccinia virus, acquired on the job site due to lack of use of Personal protective equipment.

We describe here a case of Milker's nodules with its dermatoscopy and histology features. We believe that the number of cases is greater than found in literature; as the disease is self‐limited, many do not seek medical help, making it appear relatively infrequent.

## 2 CASE REPORT

**FIGURE 1:**
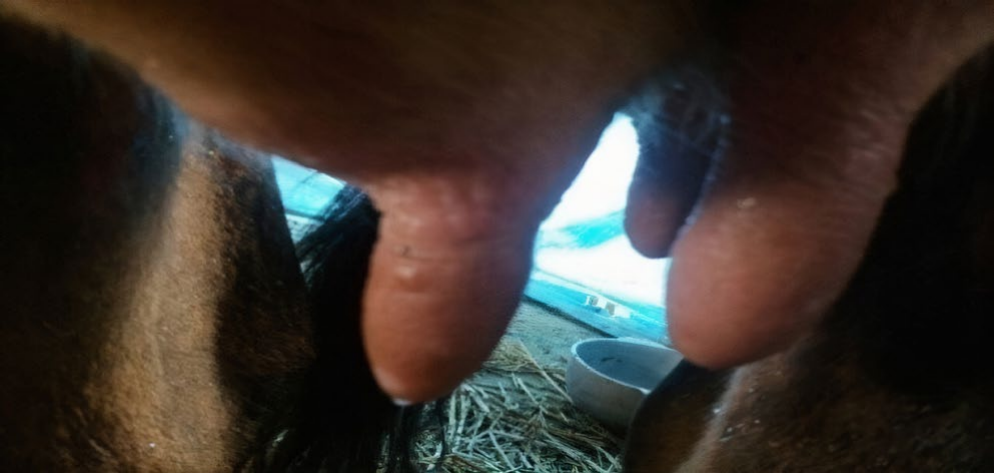
Nodules on the teats of cow

A 30‐year‐old male dairy farm worker presented with the appearance of multiple painless nodules on fingers of bilateral hand for 1 month. After 7‐10 days, he developed vesicles which ruptured followed by the formation of hemorrhagic crust. There was history of discomfort and pain over the medial aspect of right arm and forearm. He did not associate any triggering factor, denied local trauma, and/or insect bite; however, a history of having similar lesions on the teats of his cow was present (Figure 1).

**FIGURE 2:**
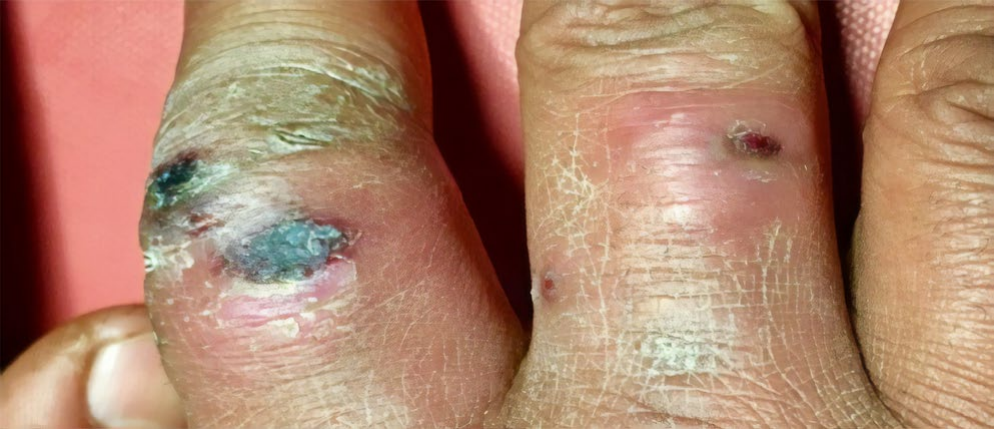
Nodules on dorsal aspect of proximal phalanges with central hemorrhagic crust and peripheral erythema

**FIGURE 3:**
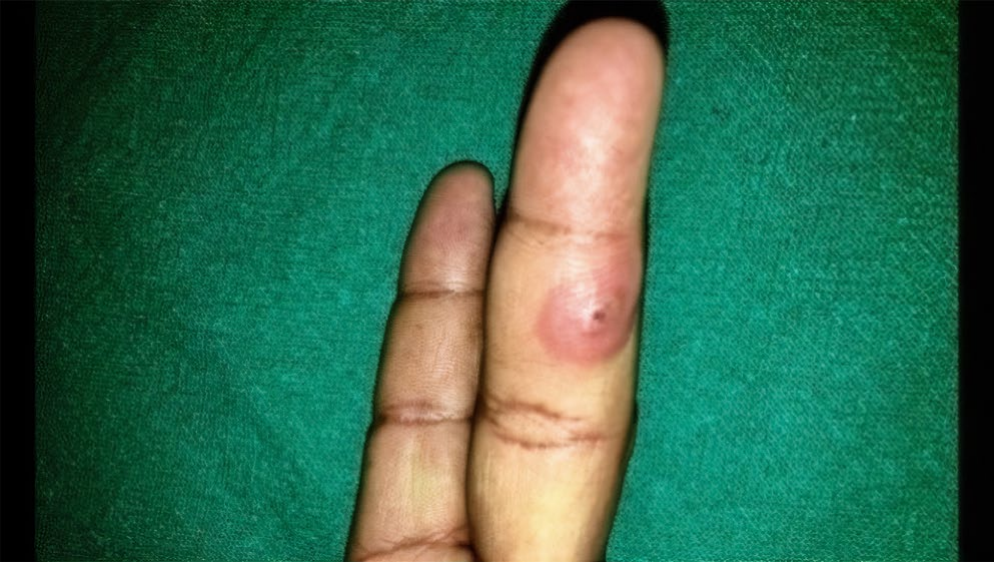
Nodule with central vesicle with peripheral erythema

Cutaneous examination revealed firm, nontender, 3‐4 nodules with central hemorrhagic crust surrounded by well to ill‐defined erythema on the phalanges of bilateral hand (Figure 2) and (Figure 3) with raised temperature and mild tenderness along the medial aspect of right arm and forearm.

**FIGURE 4:**
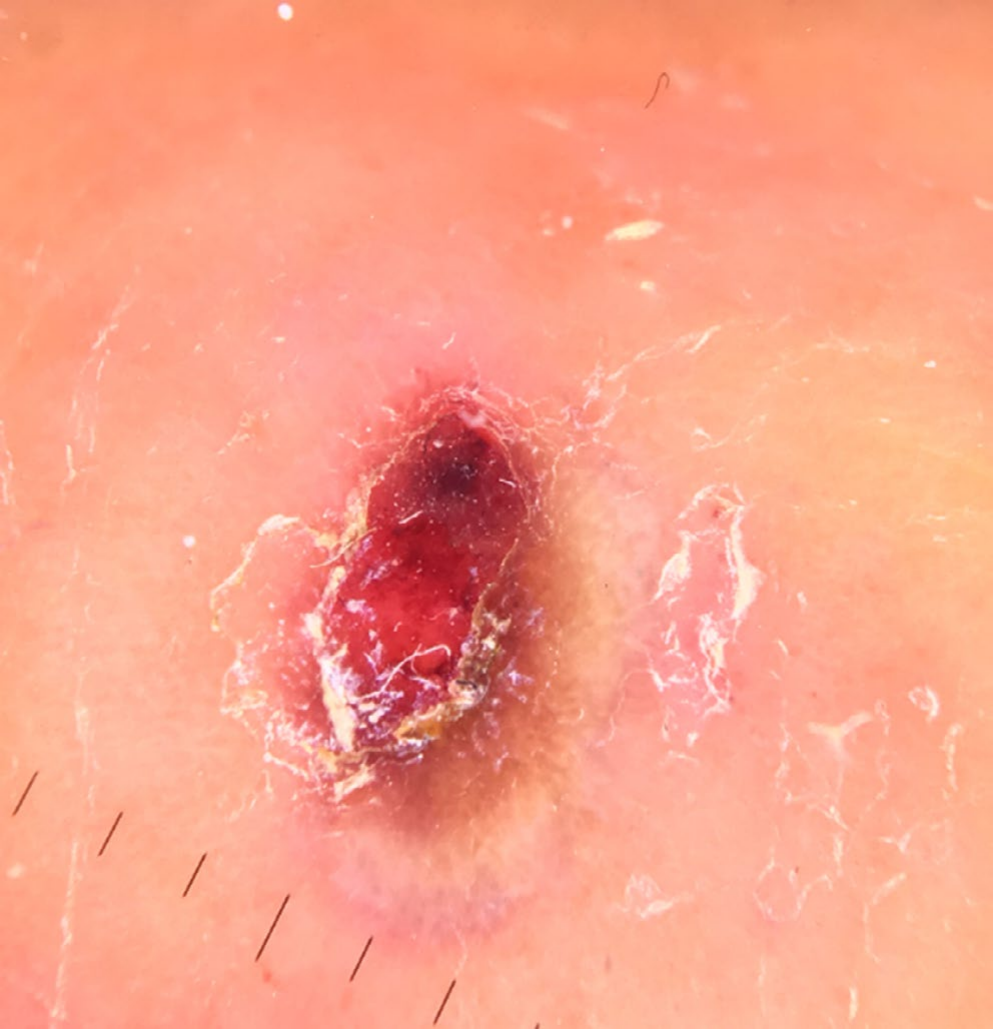
Dermoscopic Findings: an erythematous area, central ulceration, crust, yellow‐white streaks, brown dots, and structure less yellow‐whitish area partially surrounding it with erythematous ring and dot vessels; original magnification X10 (Dermlite DL3N,3Gen,Inc)

On dermatoscopy, the lesions showed an erythematous area, central ulceration, crust, yellow white streaks, brown dots, structure less whitish area partially surrounding it with erythematous ring and dot vessels (Figure 4).

**FIGURE 5:**
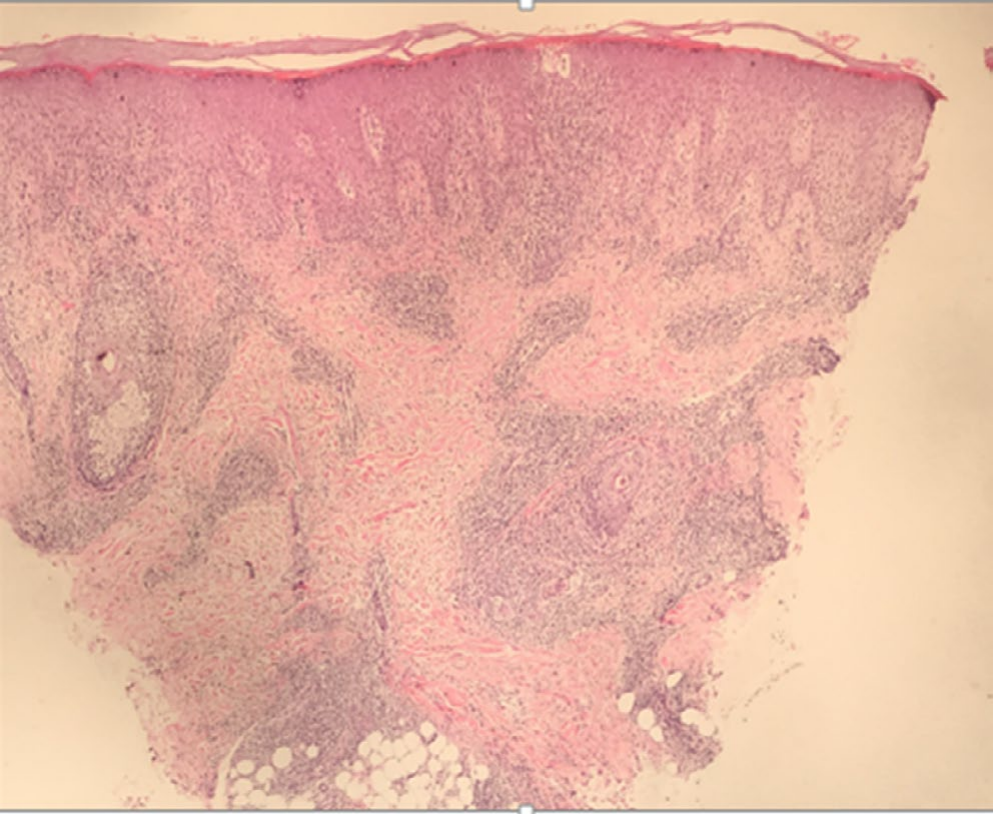
Hematoxylin and eosin stain in 4X

**FIGURE 6:**
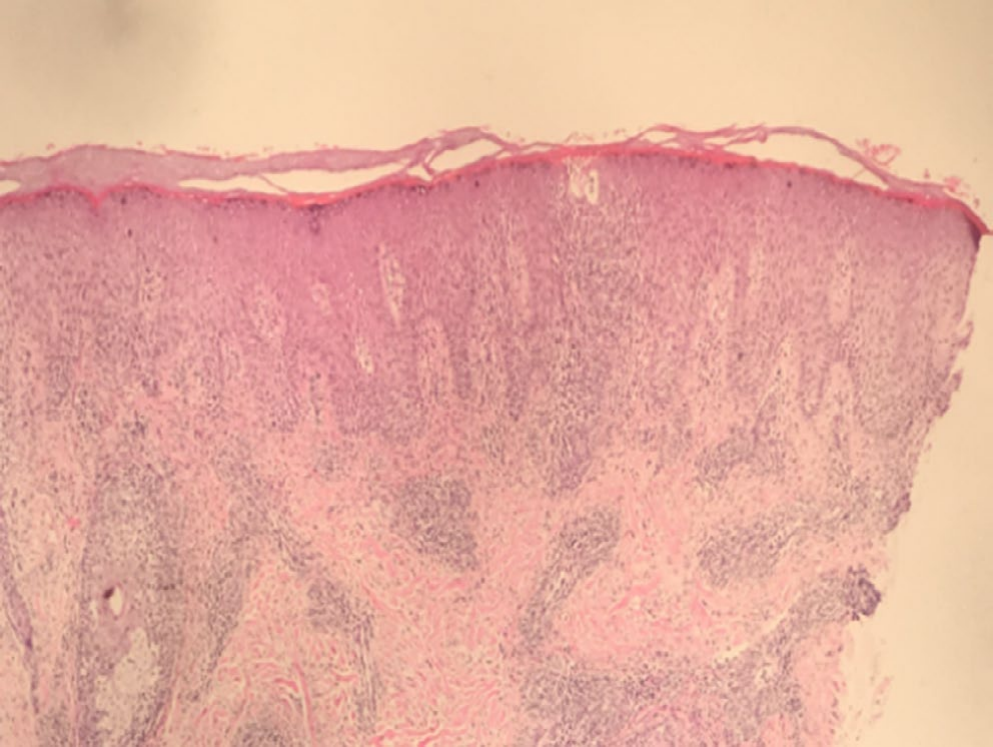
Hematoxylin and eosin stain in 10X

**FIGURE 7:**
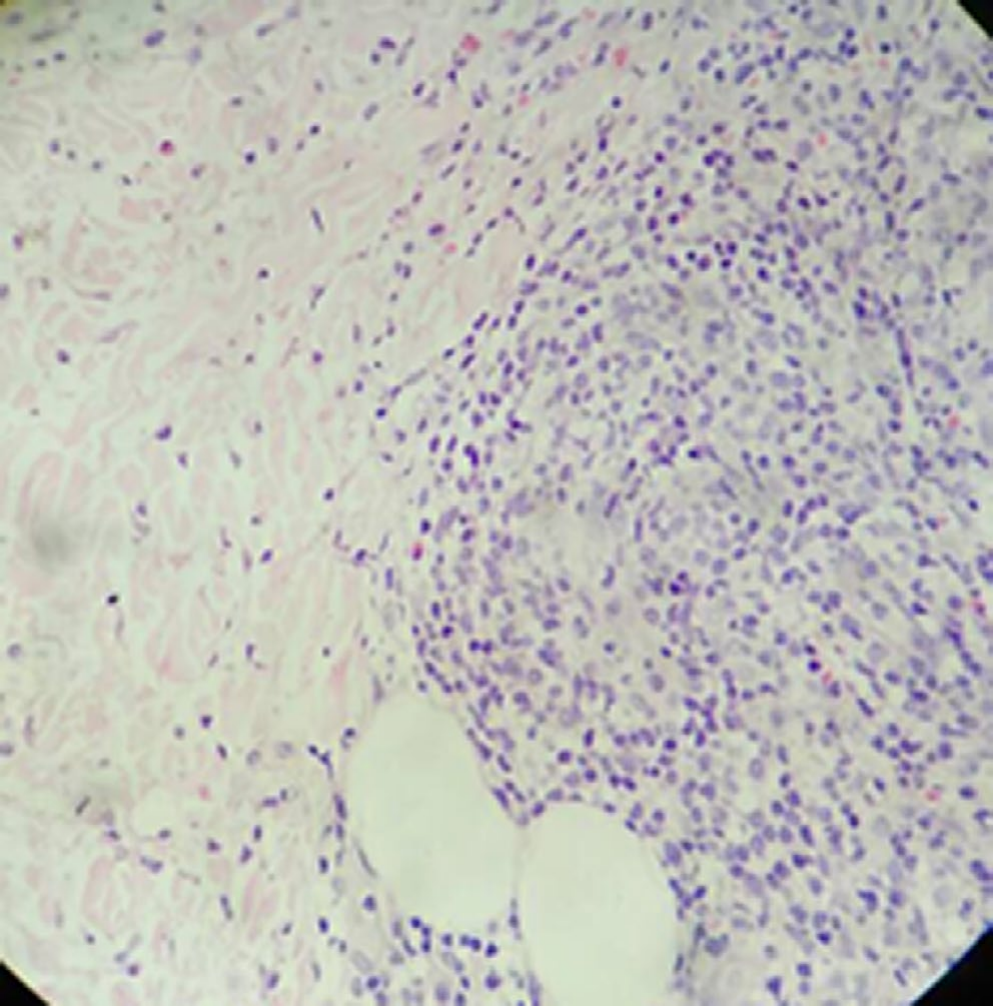
Hematoxylin and eosin stain in 40X

Histopathological examination of the skin biopsy from the nodule revealed compact hyperkeratosis with focal parakeratosis, irregular acanthosis, varying degree of spongiosis with exocytosis of lypmphocytes, and dermis revealing perivascular, periadnexal and interstitial lymphohistiocytic infiltrate extending to subcutis (Figures 5, 6, 7).

Based on history, clinical, dermatoscopy, and histopathological examination, the diagnosis of Milker's nodule with lymphangitis was made.

Counseling, reassurance, and advice regarding the use of personal protective equipment while handling infected animal was done, and the patient was managed with oral amoxicillin and calvulanic acid (625 mg three times a day) for 1‐week oral NSAID (diclofenac 100 mg/d) for pain management and topical antibiotics. All lesions were healed completely in 2‐3 weeks.

## 3 DISCUSSION

Milker's nodule is also known as pseudocowpox virus, which is caused by the Paravaccinia virus, a DNA poxvirus of the genus Parapoxvirus.^1^, ^2^ It is an universally distributed occupational viral skin disease, which occurs in persons who manage dairy cattle whether by touching infected udder or noses of cows or those who manipulate meat and/or contaminated objects.

The incubation period ranges from 5 to 15 days, and the number of lesions varies from one to five predominantly involving the hands and forearms, though it may also occur on the face.^1^ It presents as asymptomatic one or more papules developing into reddish blue, firm, painless vesicular nodule with a ring of erythema, which passes through six clinical stages, each lasting about one week. The lesions appear as erythematous macules (Erythematous maculopapular), which become target‐shaped with central ulceration and white ring, and red periphery (Target papulovesicular lesion) and then exudative nodules characterized by loss of epidermis over the center (Acute weeping nodules). The fourth phase is the formation of dry, crusted, hard, nontender nodules with darks spots on the surface (Nodular stage). These lesions became papillomatous nodules with irregular surface (Papillomatous) and finally the lesions involute without scar (Regressive).^3^

The lesions are self‐limiting and usually disappear spontaneously within 4‐6 weeks. However, the lesions may last for months and it tends to recur in immunocompromised individuals. Uncommonly, the patients may present with fever, lymphadenopathy, lymphangitis, erythema multiforme, and secondary bacterial overgrowth of the lesions.^1^

The diagnosis of Milker's nodules is mostly done by the detailed history and physical examination. Some supportive investigations such as dermoscopy and histopathology features are helpful tool for the diagnosis of this condition.

The dermoscopy findings of Milker's nodules are generally classified into four categories: Type 1 lesions shows central yellow white area and erythematous ring outside; type 2 shows orange yellow streaks in the center with violaceous erythematous base and grayish whitish streaks around and the erythematous ring outside; ulceration in the center, yellow white ring around, and erythema ring outside present in type 3; and erythema in the center or ulcer‐crusted area and yellow white ring around in type 4. Black dot and polymorphic vessels (predominated dot and comma vessels) may also be present in different type of lesions.^4^ In our case, ulceration in the center, crust and yellow white ring and erythema ring was present corresponding to the stage 3 of disease progression.

The histological examination of lesions reveals hyperkeratotosis, mild to moderate acanthosis, spongiosis, eosinophilic cytoplasm, and nuclear inclusions in the epidermis and dense lymphohistocytic infiltrates in the dermis. Depending upon the stages, some of the histologically findings may be predominant. During earlier stages namely maculopapular and target stages, vacuolization of epidermal cells with eosinophilic inclusion bodies leading to multilocular vesicles is limited to upper third of stratum Malpighi. However, throughout epidermal necrosis and massive infiltration of mononuclear cells are observed in acute weeping stage and in later stages acanthosis and vasodilation with chronic inflammatory cell infiltrate are predominant in dermis and subcutis.^5^

The confirmation of the diagnosis is made by viral isolation, starting with the tissue culture or by electron microscopy, where the virus is seen as cylindrical shape.^6^ Histogenesis and viral identification via tissue culture in bovine or Rhesus monkey kidney cells are preferred if lesions are <2 weeks however viral antigen demonstration in lesional material for older lesion.^5^ PCR is the quickest and most reliable method, which can distinguish between the various subgroups of the parapoxvirus.^7^

The patients should be managed by supportive treatment, counseling and reassurance with domestic animal pet's treatment by vets to ensure complete management.

The most common differential diagnosis is Orf, which is also a viral skin disease caused by parapoxvirus of the Poxviridae family, which is mostly diagnosed by the history of contact with ovine or caprine herds (rather than with bovines), given that they are clinically and histopathologically undistinguished.^8^ Other differential diagnoses of Milker's nodules include anthrax, atypical mycobacteriosis, and tularemia.

In our case, the classical lesions of target and nodular stage with dermoscopy and histological finding, resolution in 2‐3 weeks, lesions on teat of the cow, and the feature of lymphadenitis subsided within 1 week were quite characteristic of Milker's nodules.

## 4 CONCLUSION

The diagnosis of Milker's nodules is usually made with clinical acumen without the necessity of elaborate and costly laboratory methods. It is also important to emphasize the use of nonpermeable gloves by handling the infected cattle until complete resolution of lesions to reduce the risk of human infection.

## CONFLICT OF INTEREST

None declared.

## AUTHOR CONTRIBUTION

SA: involved in concept, idea, and editing; GPP: involved in preparation, literature review, and processing; SD: involved in histopathological report.
